# Multimodal single-cell dataset of juvenile rat neocortical interneurons—Morphology, whole-cell patch electrophysiology and histological slices

**DOI:** 10.3389/fnins.2026.1926661

**Published:** 2026-09-09

**Authors:** Maria Toledo-Rodriguez, Ying Shi, Julie Meystre, Deborah La Mendola, Rajnish Ranjan, Liviu Soltuzu, Maurizio Pezzoli, Rodrigo de Campos Perin, Henry Markram

**Affiliations:** Laboratory of Neural Microcircuitry (LNMC), École Polytechnique Fédérale de Lausanne (EPFL), Brain Mind Institute, Lausanne, Switzerland

**Keywords:** brain slice, electrophysiology, neuronal morphology, *Rattus norvegicus*, somatosensory cortex

## Abstract

This dataset provides paired single-cell morphology, whole-cell patch-clamp electrophysiology, and source histological slide imagery for juvenile rat neocortical neurons from the EPFL Laboratory of Neural Microcircuitry (LNMC, gathered by MT). The curated explorer subset spans 396 slices, with 244 3D morphological reconstructions, 641 whole-cell patch-clamp recordings, and 244 histological slide images. Recordings were acquired with a standardized current-clamp stimulus battery of 36 distinct protocols. Morphologies are stored in SWC, recordings in NWB plus decimated JSON, and slide imagery in JPEG, linked through a normalized cell identifier so that structure, function, and underlying histology can be analyzed for the same cells. The dataset is released as a FAIR^2^ Data Package with structured metadata, QUDT units, PROV-O provenance, and MLCommons Croissant conformance, intended for neuroscientists, modelers, and machine-learning researchers studying the relationship between cortical neuron morphology and intrinsic electrophysiological behavior.

## Introduction

Understanding how the intrinsic electrophysiological behavior of a neuron relates to its three-dimensional structure is a central problem in cellular and computational neuroscience. Linking the two requires datasets in which the same identified neurons are characterized both functionally, through patch-clamp recording, and structurally, through morphological reconstruction. Such paired data underpin the construction of biophysically detailed neuron models and the classification of cortical cell types (Markram et al., 2015), yet they remain comparatively scarce and are often distributed in heterogeneous, non-machine-actionable forms.

This dataset addresses that gap for neocortical neurons studied at the EPFL Laboratory of Neural Microcircuitry (LNMC), gathered by MT (Maria Toledo-Rodriguez). It combines a large library of standardized whole-cell patch-clamp recordings with single-cell morphological reconstructions and the source histological slide imagery, recovered and harmonized from the original LNMC laboratory archives. A standardized current-clamp stimulus battery comprising 36 distinct protocols was applied across all recorded cells, enabling consistent comparison of intrinsic membrane properties, action-potential generation, and firing behavior across cells of the juvenile rat somatosensory cortex.

The advent of machine learning in neuroscience has raised the bar for data reuse: training pipelines require curated, well-documented, standardized resources with machine-readable provenance, computable licenses, and unambiguous dataset boundaries. To meet these needs, the recordings are preserved in the community-standard NWB (icephys) format (Rübel et al., 2022) and the morphologies in the SWC standard, and both are indexed into tidy, queryable Parquet tables linked by a normalized cell identifier.

The result is provided as a FAIR^2^ Data Package—Findable, Accessible, Interoperable, Reusable, AI-ready, and Responsibly governed—with structured metadata conforming to the MLCommons Croissant specification (MLCommons, 2024), QUDT units, and PROV-O provenance following the FAIR principles (Wilkinson et al., 2016). This article documents the dataset's design, contents, methods, and quality, and provides a visual overview of the morphological and electrophysiological data.

## Methods summary

### Study design

This study characterizes the intrinsic electrophysiological and morphological properties of cortical interneurons in the juvenile rat somatosensory cortex. Experiments were conducted on Wistar rats sampled within the post-natal day 11 to 16 developmental window, following the acute-slice and intracellular-labeling preparation established by Wang et al. (2002) for nest basket cells in the developing somatosensory cortex. Every recorded neuron was acquired in a single integrated experiment that aimed at pairing whole-cell patch-clamp recording with intracellular biocytin labeling, so that the same cell yields both a quantitative electrophysiological fingerprint and a three-dimensional morphological reconstruction. Each recording was characterized by a 61-feature quantitative electrophysiological descriptor covering passive membrane properties, action-potential shape, and firing dynamics (Toledo-Rodriguez et al., 2004), and each reconstructed neuron was assigned to a cortical interneuron class such as large, small, or nest basket cell (LBC, SBC, NBC), so that electrophysiological and morphological identities are available for the same cell.

### Animals

Experimental subjects were juvenile Wistar rats (*Rattus norvegicus*). Cross-linking the curated bundle against the project's MT_SingleCellData ledger by experiment date matched 370 of the 396 kept slices to demographically annotated records (the remaining 26 are 2005 recordings post-dating the ledger snapshot). Within the matched set the cohort spans post-natal day 11 to 16 (P11–P16) with the bulk between P13 and P16: P11 = 3 (0.8 %), P12 = 13 (3.5 %), P13 = 85 (23.0 %), P14 = 114 (30.8 %), P15 = 77 (20.8 %), P16 = 78 (21.1 %). This developmental window was chosen so that the cortical microcircuit is sufficiently mature for stable patch-clamp recording while tissue viability during acute slicing remains high (Wang et al., 2002). Sex was recorded for 350 of the matched cells (M = 134, F = 216). All animal procedures were conducted in accordance with Swiss national legislation and the institutional guidelines of the École Polytechnique Fédérale de Lausanne (EPFL). Animals were rapidly decapitated and the brain was carefully removed and immediately placed in ice-cold artificial cerebrospinal fluid (ACSF) to minimize post-mortem deterioration of cortical tissue prior to sectioning.

### Slice preparation

Acute sagittal slices of 300 μm thickness were cut from each brain on a vibratome, using either an HR2 instrument (Sigmann Elektronik, Heidelberg, Germany) or a Leica Microsystems vibratome (Wetzlar, Germany). Slices contained the full thickness of somatosensory cortex spanning layers I to VI, preserving the intracortical microcircuit around each recorded neuron (Wang et al., 2002). Following sectioning, slices were incubated in oxygenated ACSF at 37 °C for 30–60 min and were subsequently maintained at room temperature until the time of recording. For recording, individual slices were transferred to a chamber perfused with oxygenated ACSF and held at 34 °C ± 1 °C. Neurons in the somatosensory cortex were visualized by infrared differential interference contrast (IR-DIC) videomicroscopy, using a VX55 camera (Till Photonics, Germany) mounted on an upright BX51WI microscope (Olympus, Japan), and were targeted on the basis of their somatic morphology and laminar position.

### Slice preparation solution

Slices were prepared in artificial cerebrospinal fluid (ACSF) containing, in millimolar concentrations, 125 NaCl, 25 NaHCO_3_, 2.5 KCl, 1.25 NaH_2_PO_4_, 2 CaCl_2_, 1 MgCl_2_, and 25 D-glucose, bubbled with a mixture of 95% O_2_ and 5% CO_2_. The carbogen-equilibrated ACSF provided the oxygenated, buffered bathing medium maintained around the slice throughout recording. Unless otherwise specified, chemicals were obtained from Sigma-Aldrich (St. Louis, MO) or Merck (Darmstadt, Germany).

### Electrophysiological recordings

Patch pipettes were fabricated from borosilicate glass using a Flaming/Brown micropipette puller, model P-97 (Sutter Instruments, Novato, CA), and were pulled to tip resistances of 3 to 8 MΩ. Pipettes were filled with the intracellular biocytin-containing solution and used to establish whole-cell patch-clamp recording on visually targeted neurons (Wang et al., 2002). Single-cell somatic recordings were performed in current-clamp mode using Axopatch 200B or Multiclamp 700B amplifiers (Molecular Devices, San Jose, CA), with recording temperature maintained at 34 °C ± 1 °C. Series resistance and seal stability were monitored continuously throughout the recording. Data acquisition was performed via an ITC-18 or ITC-1600 board (Instrutech, Port Washington, NY) connected to a computer running custom routines under IgorPro (WaveMetrics, Portland, OR). Signals were sampled at 5–10 kHz and low-pass filtered at 2 kHz using a Bessel filter, defining the temporal resolution and bandwidth of every recorded trace. After recording, slices were fixed in a paraformaldehyde and glutaraldehyde-containing fixative to preserve morphology for histological processing and three-dimensional reconstruction (Wang et al., 2002).

### Electrophysiological recording solutions

Slices were continuously superfused with artificial cerebrospinal fluid (ACSF) containing, in millimolar concentrations, 125 NaCl, 25 NaHCO_3_, 2.5 KCl, 1.25 NaH_2_PO_4_, 2 CaCl_2_, 1 MgCl_2_, and 25 D-glucose, bubbled with a mixture of 95% O_2_ and 5% CO_2_. The carbogen-equilibrated ACSF provided the oxygenated, buffered bathing medium maintained around the slice throughout recording. Unless otherwise specified, chemicals were obtained from Sigma-Aldrich (St. Louis, MO) or Merck (Darmstadt, Germany). The intracellular pipette solution contained, in millimolar concentrations, 110 K-gluconate, 10 KCl, 4 ATP-Mg, 10 phosphocreatine, 0.3 GTP, 10 HEPES, and 13 biocytin, following Wang et al. (2002). The solution was adjusted to pH 7.3–7.4 using 5 M KOH, and osmolarity was adjusted to 290–300 mOsm using D-mannitol at 25–35 mM. Biocytin diffused passively into the patched neuron during recording, filling the soma, dendrites, axons, and putative synaptic boutons; the labeled cell was later recovered histologically using avidin–biotin complex amplification with a diaminobenzidine chromogenic reaction so that the same neuron could be reconstructed in three dimensions. Membrane potential values were not corrected for the liquid junction potential, which was approximately −14 mV.

### Stimulus protocol

Each cell was subjected to a standardized current-clamp stimulus battery of 36 distinct protocols applied identically across recordings (Toledo-Rodriguez et al., 2004). The battery covers electrode and recording-stability checks, subthreshold passive properties, hyperpolarization/after hyperpolarization behavior, action-potential threshold and waveform, suprathreshold firing patterns, spike-recovery and refractoriness, and resonance/noise characterization, so that the resulting 61-feature quantitative electrophysiological descriptor is comparable across cells (Toledo-Rodriguez et al., 2004; Wang et al., 2002). Within the curated explorer subset of 641 recordings, the most-used protocols by sweep count are IDRest, IDthresh, IV, APWaveform and sAHP. The full grouping is: Electrode and recording-stability checks: Test_eCode; VacuumPulses; Spontaneous; SpontAPs. Subthreshold passive properties (input resistance, current–voltage): IRrest; IRdepol; IRhyperpol; IV; IV_Test; ADHPrest. Hyperpolarization-activated/after hyperpolarization: ADHPdepol; ADHPhyperpol; IDdepol; IDhyperpol; sAHP. Action-potential threshold and waveform: IDthresh; APThreshold; APWaveform; APDrop; HighResThResp. Suprathreshold firing patterns and adaptation: IDRest; C1_HP_1sec; C1_HP_0.5sec; C1step_1sec; C1step_ag; C1step_highres; Delta; maria-STEP. Spike-recovery / refractoriness: SpikeRec; SpikeRec_Kv1.1; SpikeRec_Ih. Resonance/impedance and noise stimuli: SineSpec; Truenoise; NoisePP; NoiseSpiking. Suprathreshold depolarising current steps (IDRest and the C1 step family) were used to classify firing phenotype—accommodating, non-accommodating, stuttering, delayed-firing, or burst-firing (Wang et al., 2002)—and these classifications were used to assign each cell to a cortical interneuron subclass. From each recording a 61-feature quantitative electrophysiological descriptor was extracted, after Toledo-Rodriguez et al. (2004), covering passive membrane properties, action-potential waveform features, and firing dynamics including delay to first spike, initial burst interval, adaptation index, steady-state firing rate, and coefficient of variation of firing.

### Morphological reconstruction and analysis

Recorded neurons were filled with biocytin during the whole-cell recording and, following slice fixation in a paraformaldehyde and glutaraldehyde-containing fixative, were recovered histologically using avidin–biotin complex (ABC) amplification with a diaminobenzidine (DAB) chromogenic reaction to produce stable staining suitable for light microscopy (Wang et al., 2002). Filled neurons were then reconstructed in three dimensions using Neurolucida (MicroBrightField) under light microscopy on an Olympus BX51 microscope fitted with a 100 × /1.3 oil-immersion objective (Olympus), following the detailed procedure described previously (Wang et al., 2004). The reconstructed morphologies are distributed as SWC-format neuron trees under swc/ < slice>/ < id>.swc.

## Data overview

### Quantitative summary of the dataset

The curated explorer subset of the dataset provides paired single-cell morphology, whole-cell patch-clamp electrophysiology, and source histological slice imagery for neocortical neurons of juvenile rat. The subset is assembled into a self-contained explorer bundle with one record per slice (records.json), per-modality artifacts (3D morphology SWC, decimated sweep JSON, slide imagery). Across the bundle the dataset spans 396 slices, 244 3D reconstructions, 641 whole-cell recordings, and 244 histological slide images.

### Dataset composition

The curated explorer bundle comprises five canonical asset groups linked to one record per slice—approximately 3.92 million primary records when SWC nodes and decimated ephys sweeps are counted together:

- Slices and per-slice records: 396 curated slices carried in records.json; each record is one slice with its modality presence flags and per-item lists.
- Cell morphology reconstructions: 244 SWC files under swc/ < slice>/ < id>.swc-−3,886,425 SWC nodes across all reconstructions, with median 15,610 nodes per cell (range 1,944–39,553). Per-compartment node totals: soma (7,531), axon (3,113,419), basal dendrite (688,780), apical dendrite (76,695).
- Whole-cell patch-clamp recordings: 641 recordings under sweeps/ < slice>/ < stem>.json-−30,563 decimated sweeps in total, with median 48 sweeps per recording (range 1–48). Each sweep JSON carries protocol, sampling rate, full sample count, and min/max-decimated points for plotting; the raw NWB files for the same recordings live in the source package under assets/nwb/.
- Histological slide images: 244 slide images under images/ < slice>/ < file>, with the annotated 10 × JPG preferred when available; per-protocol sweep family thumbnails (15,933 PNG files, ~97 MB) live under thumbnails/ < slice>/ephys/ < stem>/ and drive the ephys section's preview grid.

### Data structure and formats

The bundle is organized as a self-contained tree (records.json drives a multi-modal viewer; sections.json declares the three modality sections; meta_fields.json declares the scalar fields shown under the viewer). Asset payloads are SWC (Cannon et al., 1998) for morphology, min/max-decimated JSON sweeps for electrophysiology (the canonical NWB files remain in the source FAIR^2^ package), and JPEG/PNG for slide imagery. Total bundle footprint: ~2.0 GB (310 MB SWC, 1.0 GB sweep JSON, 550 MB slide images, 97 MB sweep thumbnails). All artifact paths inside records.json are bundle-relative—segment-encode “#” characters in slice ids when constructing artifact URLs (e.g., “C100100#6” → “C100100%236”).

### Feature and attribute space

Per-slice records expose modality-presence flags (has_morphology, has_ephys, has_slide), the representative item's QC fields per modality (qc_score, n_nodes/soma_present/has_axon/has_basal/has_apical for morphology; rmp_mv/rmp_drift_mv/baseline_noise_mv/detected_spike_count/max_depolarization_mv/clipping_fraction for ephys), and per-item JSON arrays (morph_reconstructions, ephys_recordings, slide_thumbnails) addressable by the viewer's item selector. Combined cross-modal QC is summarized by “combined_qc_score.”

### Categorical and taxonomic diversity

- Specie: juvenile rat (*Rattus norvegicus*, Wistar). Per-slice labels are carried in the source package's fair2.json (cellComposition) rather than in the explorer records; the expanded methods (Methods Summary) describe acquisition protocols.
- Morphological compartment types (4 SWC type codes 1–4): soma, axon, basal dendrite, apical dendrite. Axon dominates reconstruction size at 80.1% of all SWC nodes; basal dendrite contributes 17.7%, apical 2.0%, soma the remainder.
- Stimulus protocols (36 distinct types across 30,563 decimated sweeps in the bundle): a standardized adaptive stimulus battery covering electrode/cell characterization, subthreshold and suprathreshold steps, and resonance/noise stimuli. The 10 most-represented protocols are IDRest (4103), IDthresh (3573), IV (3364), APWaveform (3053), sAHP (2260), Test_eCode (2234), C1_HP_1sec (1882), SpikeRec (840), C1step_ag (794), ADHPdepol (726).
- Brain region: primary somatosensory cortex (juvenile rat S1, including the hindlimb representation;). Per-cell anatomical annotations live in the source package's cellComposition records.

### Data completeness

- Morphology nodes: every reconstruction is a complete point-and-radius tree with full parent linkage (median 15,610 nodes per neuron; range 1,944–39,553). Soma is present in all 244/244 reconstructions, axon in 238, basal dendrite in 243; apical dendrite is present in 28 (consistent with the cohort being dominated by cortical interneuron classes).
- Electrophysiology: every recording carries the standardized stimulus battery (median 48 decimated sweeps per recording, with a per-recording cap of 48 in the bundle); the canonical NWB files in the source package retain the full raw acquisition.
- Cross-modal coverage across the 396 curated slices—all counts in this bullet are per slice, not per cell: 99 slices have all three modalities (morphology + ephys + slide), 53 have morphology + ephys but no slide image, 10 have morphology + slide, 113 have ephys + slide, 99 are ephys-only, 22 are slide-only, and 0 are morphology-only. In total 162 slices carry morphology, 364 carry ephys, and 244 carry slide imagery. Morphology and ephys therefore co-occur in 152 slices (99 + 53), keeping in mind that a single slice can contribute several recorded cells, and not every cell recorded on a slice was reconstructed.
- No imputation or interpolation was applied; raw signals are preserved in the source-package NWB and SWC files, and the explorer bundle's sweep JSON is min/max-decimated only for plotting (full acquisition retained in the source package).

## Data quality and curation criteria

Every released item carries per-check quality flags and a summary score. The criteria below are the ones actually applied; the flags themselves ship with the data so users can impose stricter thresholds of their own.

Electrophysiology—per-recording checks. Four checks are evaluated on the representative recording of each of the 364 slices carrying electrophysiology: resting membrane potential within −80 mV ≤ RMP ≤ −50 mV (10/364 flagged); RMP drift of at most 8 mV in absolute value over the recording (78/364 flagged); baseline noise at most 2.5 mV RMS (151/364 flagged); and signal clipping below threshold (0/364 flagged, maximum observed clipped-sample fraction 0.0036). ephys_qc_score is the fraction of these four checks passed, so it takes the values 1.00 (176 slices), 0.75 (139), 0.50 (47), and 0.25 (2). Input resistance was not derivable from the source archives—no per-sweep stimulus amplitudes were stored—and is therefore not evaluated and not part of the score.

Morphology—per-reconstruction checks. Four checks are evaluated on each of the 244 reconstructions: soma present (244/244), axon present (238/244), basal dendrite present (243/244), and tree integrity, i.e., a complete point-and-radius tree with full parent linkage (244/244). morph_qc_score is the fraction passed: 237 reconstructions score 1.00 and 7 score 0.75—six lacking a reconstructed axon (C170501A2, C190101B2, C231001B1, C230501A1, C230501A2, C230501A4) and one lacking a basal dendrite (C010700A). Apical-dendrite absence is deliberately not scored as a defect: only 28/244 reconstructions have an apical dendrite, which is the expected phenotype for a cohort dominated by basket-cell-class interneurons rather than a reconstruction failure.

The combined_qc_score is the mean of the available per-modality scores, and is undefined for the 22 slide-only slices. Across the 374 scored slices it takes the values 1.00 (185), 0.875 (50), 0.75 (105), 0.50 (32), and 0.25 (2).

Curation and inclusion process: Morphologies were processed through the Open Brain Institute morphology-workflows Curate phase (14 stages: marker extraction, neurite checks, sanitization, reentering, orientation and alignment, error detection, and resampling). Of 251 input Neurolucida reconstructions, 247 passed; four were dropped at the Sanitize stage with a zero-length root section that the tool cannot repair unambiguously (C130301C, mtC141200C_idB, mtC151200D_idA, mtC181001A_idB). The DetectErrors stage flagged 2,979 geometric issues across seven types in 177 of the 247 passing cells-−2,146 overlapping points, 657 fat ends, 165 z-range excursions, 4 z-jumps, 3 multifurcations, 2 dangling sections, and 2 narrow starts. These are flagged, not repaired and not excluded: overlapping points are digitizer click jitter that resampling absorbs, and the remainder are recorded per cell so that users who need pristine topology can filter on them. On the electrophysiology side the source archives delivered 793 metadata rows covering 635 unique recordings, because 158 cells were supplied twice across the archive set; after deduplication and reconciliation against the canonical asset inventory the released set is 641 recordings, each backed by one canonical NWB file.

Outlier and exclusion policy: Quality scores are advisory and were not used as an inclusion filter—no recording and no reconstruction was excluded from this dataset on the basis of a quality score or an outlier criterion. The only exclusions applied were structural: reconstructions that the curation pipeline could not process (the four above), and duplicate records. Flagged items are retained deliberately, because a recording with, say, elevated baseline noise remains usable for many analyses and the appropriate threshold depends on the question being asked. Users who wish to work with a stricter subset can reproduce one directly from the released per-check flags—for example, requiring ephys_qc_score = = 1.0 yields 176 recordings and combined_qc_score ≥ 0.875 yields 235 slices.

### Notable patterns

- Axonal arbor dominates reconstruction size, accounting for 80.1% of all morphology nodes (3,113,419 of 3,886,425), consistent with the dense local axonal collaterals characteristic of cortical interneurons (LBC, SBC, NBC). Apical dendrite is present in only 28/244 reconstructions, reflecting the predominance of basket-cell-class neurons over pyramidal cells in this cohort.
- Recording sampling rates are protocol-dependent. Most sweeps were acquired at 4 kHz (19,084 sweeps), with higher rates used for fast action-potential-shape protocols (50–100 kHz, 3,112 sweeps) and lower rates for long-duration spontaneous and ramp protocols.
- All acquisition signals are recorded in current-clamp configuration and stored in millivolts in the explorer bundle (volts in the source package's NWB files).

## FAIR^2^ compliance certification

### Overall FAIR^2^ badge assessment

Compliant—the dataset meets the FAIR^2^ criteria. Findability is established through a registered DOI (10.71728/senscience.bt07-sboc) and a structured data package carrying cell composition records for every kept slice. Accessibility is provided via the self-contained explorer bundle, a web-renderable viewer (MultiModalInspector reads records.json directly), and the source FAIR^2^ package's NWB and parquet distributions. Interoperability is supported by community-standard formats (NWB for raw electrophysiology, SWC for morphology, JPEG for slide imagery, JSON-LD for metadata). Reusability is supported by the ODC-By v1.0 license (Open Data Commons, 2017). The FAIR^2^ assessment criteria and supporting evidence are summarized in Table 1.

**Table 1:** FAIR^2^ compliance assessment criteria and supporting evidence for the curated multimodal neuronal dataset.

Criteria	Details
Findability (F)	
F1. Unique identifier	The dataset is assigned a DOI (10.71728/senscience.bt07-sboc) registered with DataCite for global traceability and citability.
F2. Metadata	Metadata includes title, creators and affiliation (EPFL LNMC), abstract, species (*Rattus norvegicus*), brain region (somatosensory/cerebral cortex), variables across four tables, and the data-collection and extraction methods.
F3. Metadata includes data identifiers	The DOI is embedded in the metadata, linking the metadata record to the dataset.
F4. Searchable metadata	Metadata is exposed as schema.org/Dataset (JSON-LD) and as an MLCommons Croissant 1.1 record, indexable by Google Dataset Search.
Accessibility (A)	
A1. Open access	Released under the Open Data Commons Attribution License v1.0 (ODC-By v1.0) license, permitting reuse, redistribution, and modification with attribution.
A2. Long-term access	Canonical SWC and NWB assets are preserved within the package alongside the derived tables.
API access	Structured metadata and downloadable components are exposed via the MLCommons mlcroissant API and the FAIR^2^ portal data explorer.
Interoperability (I)	
I1. Standardized formats	Tabular data in Parquet; morphologies in SWC; electrophysiology in NWB (HDF5); semantic metadata in JSON-LD.
I2. Controlled vocabularies	Units standardized with QUDT (micrometers for morphology coordinates). Fields annotated with SKOS mappings to schema.org, NCIT, UBERON, and PATO. Metadata schema based on schema.org, Croissant, and PROV-O.
I3. Cross-platform integration	Conforms to MLCommons Croissant 1.1 for AI workflows; NWB and SWC are community standards for the neurophysiology and neuromorphology domains.
Reusability (R)	
R1. Comprehensive documentation	A variable-level data dictionary describes every column across the four tables with definitions, units, and method provenance.
R1.1. License	ODC-By v1.0, permitting reuse, redistribution, and modification with attribution.
R1.2. Detailed provenance	Each table and field is linked via PROV-O to the extraction scripts (extract_morphology.py, extract_ephys.py) and the source archives.
R1.3. Domain-relevant standards	Morphologies follow the SWC standard; electrophysiology follows the NWB:N icephys standard.
Versioning and updates	Version 1.0.0 with embedded version metadata.
AI-Readiness (AIR)	
Structured for machine learning	Tidy tabular Parquet with consistent identifiers and a morphology↔electrophysiology join key, suitable for ML ingestion.
Scalable	~3.9M morphology nodes and ~195K acquisition sweeps, indexed for query while raw arrays remain in the canonical NWB/SWC files.
Training and validation sets	Natural partitioning by modality (morphology vs. electrophysiology) and by cell supports train/validation/test splits.
Responsible AI (RAI)	
Ethical standards and misuse	Intended for neuroscience research on cortical neuron structure and intrinsic electrophysiology.
Biases in the dataset	Morphology subjects and electrophysiology subjects are *Rattus norvegicus*. Coverage reflects the LNMC project MT sampling and is not a population-representative survey.
Data privacy and security	No human personal data. All subjects are non-human animals (*Rattus norvegicus*); contributor names appear in a standard scientific-attribution context.
Data provenance and accountability	Field-level provenance is encoded with PROV-O and documented in the data dictionary.
Transparency and reporting	Extraction methods and known limitations are documented; an automated integrity assessment scored 0.92.
Human-in-the-Loop (HITL) considerations	Source reconstructions and recordings were produced by the LNMC; FAIR^2^ packaging was performed by an automated wrangling agent with documented steps.

### Visual overview

The following figures provide a visual overview of the curated explorer bundle across its three modalities—morphology, electrophysiology, and histological slide imagery—covering dataset dimensions, morphological composition, representative reconstructions, the standardized stimulus battery, example whole-cell traces, and the cross-modal coverage of the kept cohort.

**Figure 1:**
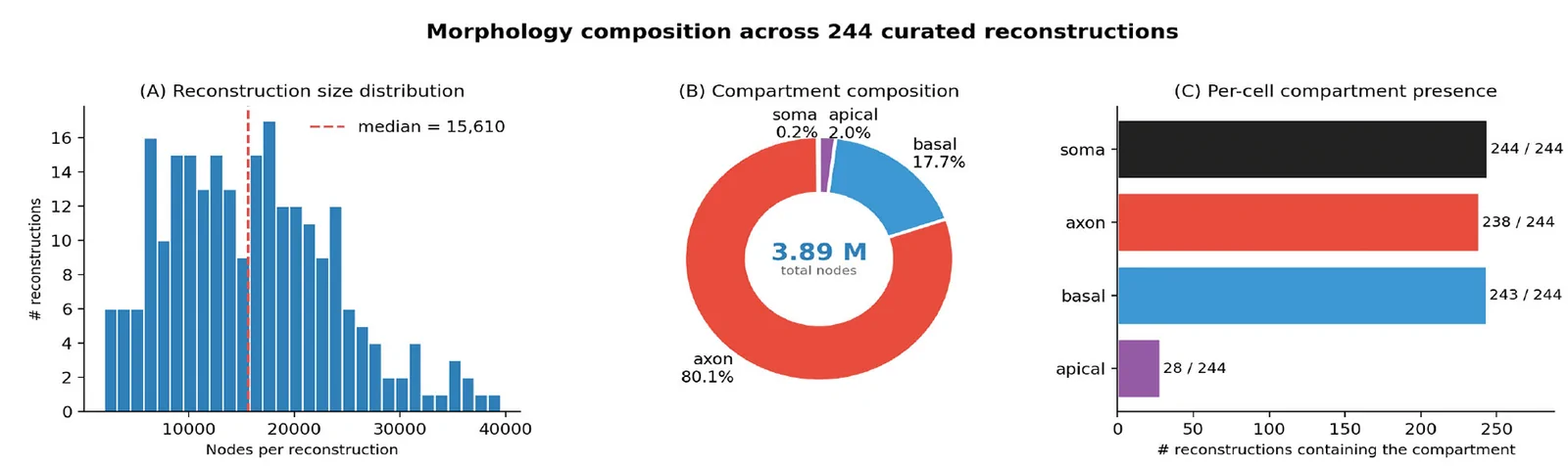
Morphology composition across the 244 reconstructions in the curated bundle. **(A)** Distribution of nodes per reconstruction (median 15,610; range 1,944–39,553). **(B)** Compartment composition of the 3.89 M total SWC nodes: axon 80.1%, basal dendrite 17.7%, apical dendrite 2.0%, soma the remainder. **(C)** Per-cell compartment presence: soma 244/244, axon 238/244, basal 243/244, apical 28/244.

The structural composition of the reconstructions is characterized in Figure 1, summarizing reconstruction size, compartment composition, and per-cell compartment presence across the curated SWC set.

**Figure 2:**
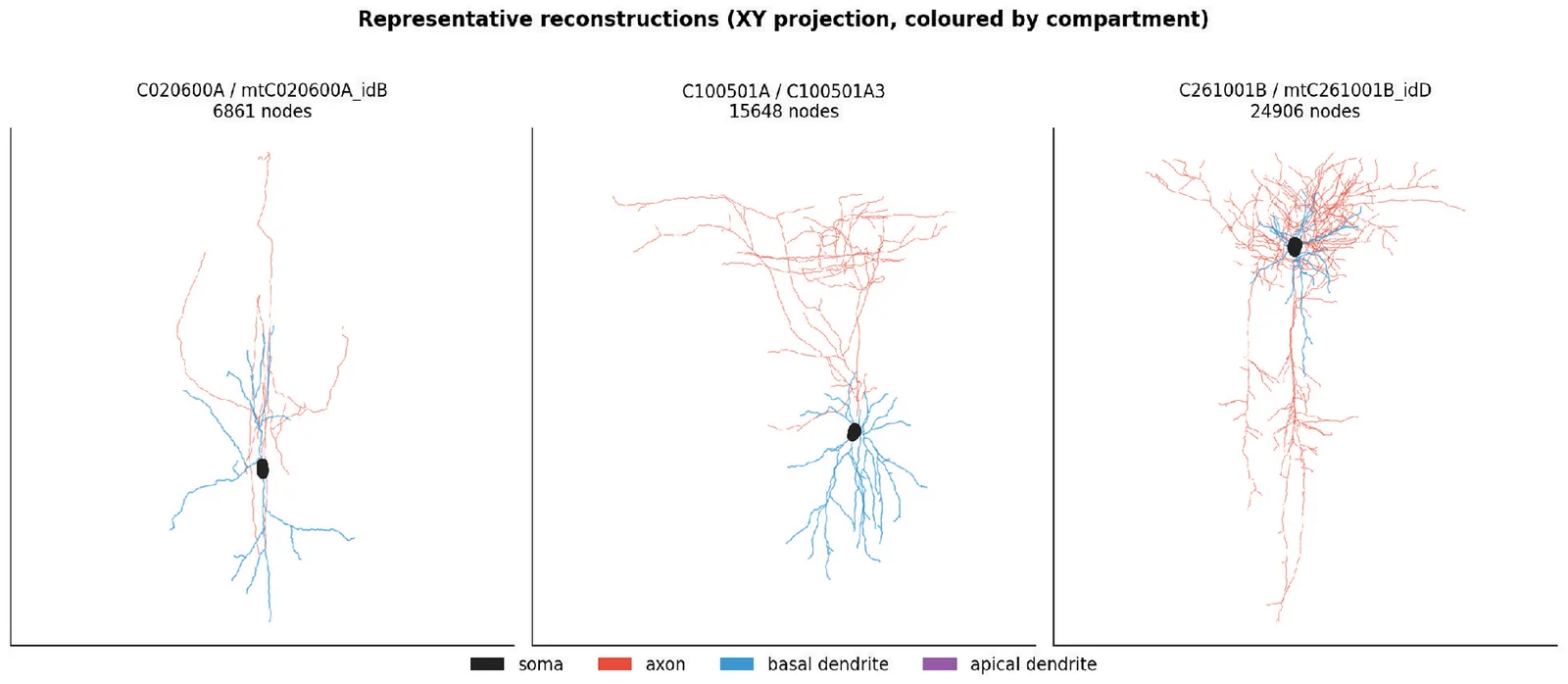
Representative neuronal reconstructions (XY projection) colored by SWC compartment type (soma, axon, basal dendrite, apical dendrite). Three reconstructions are shown spanning small, medium, and large total node counts in the curated bundle.

Representative reconstructions spanning a range of sizes are shown in Figure 2, illustrating the soma, axonal, and dendritic compartments captured by the SWC trees.

**Figure 3:**
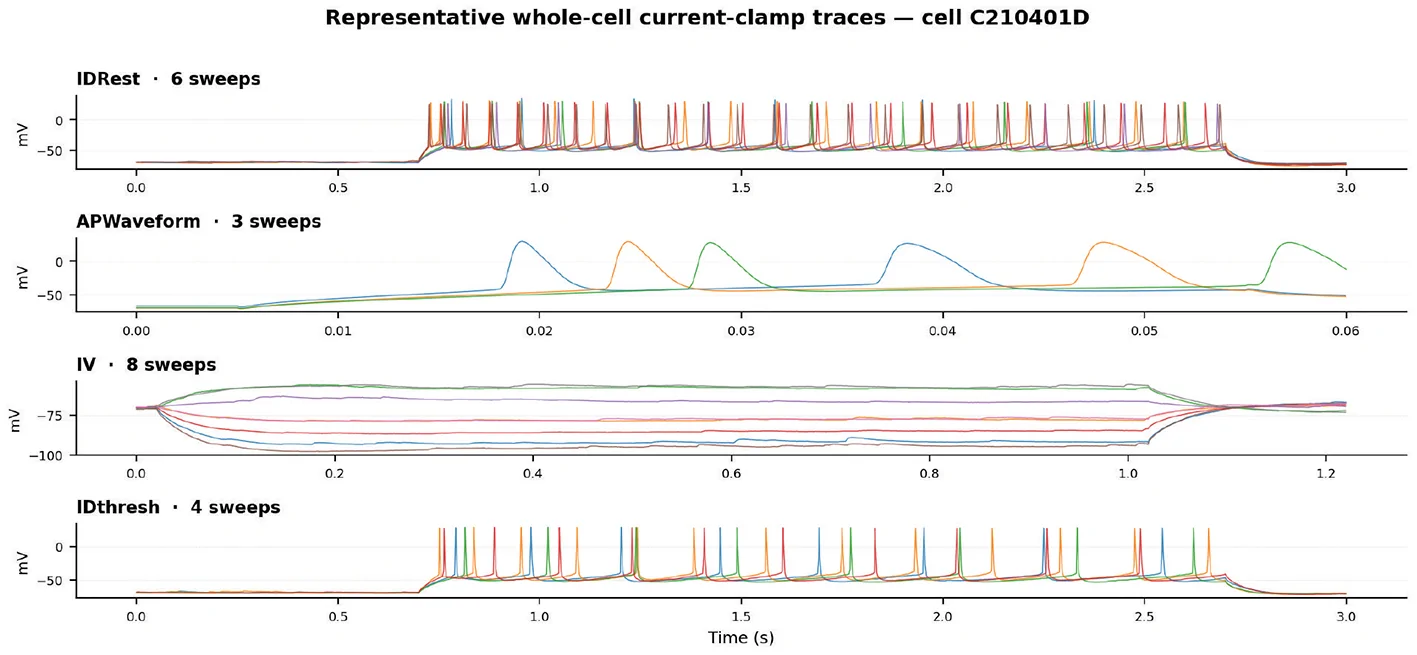
Representative whole-cell current-clamp traces from a single recording (cell C210401D) in the curated bundle, drawn across four distinct stimulus protocols (one row per protocol, up to eight sweeps per row, each sweep in a different color). The IV panel is shown as a subthreshold family (no spikes) to illustrate the passive-response regime; the IDRest, APWaveform, and IDthresh panels illustrate the suprathreshold and threshold-crossing regimes.

Representative voltage responses from a single cell are shown in Figure 3, illustrating the diversity of intrinsic behaviors probed by the standardized stimulus battery.

**Figure 4:**
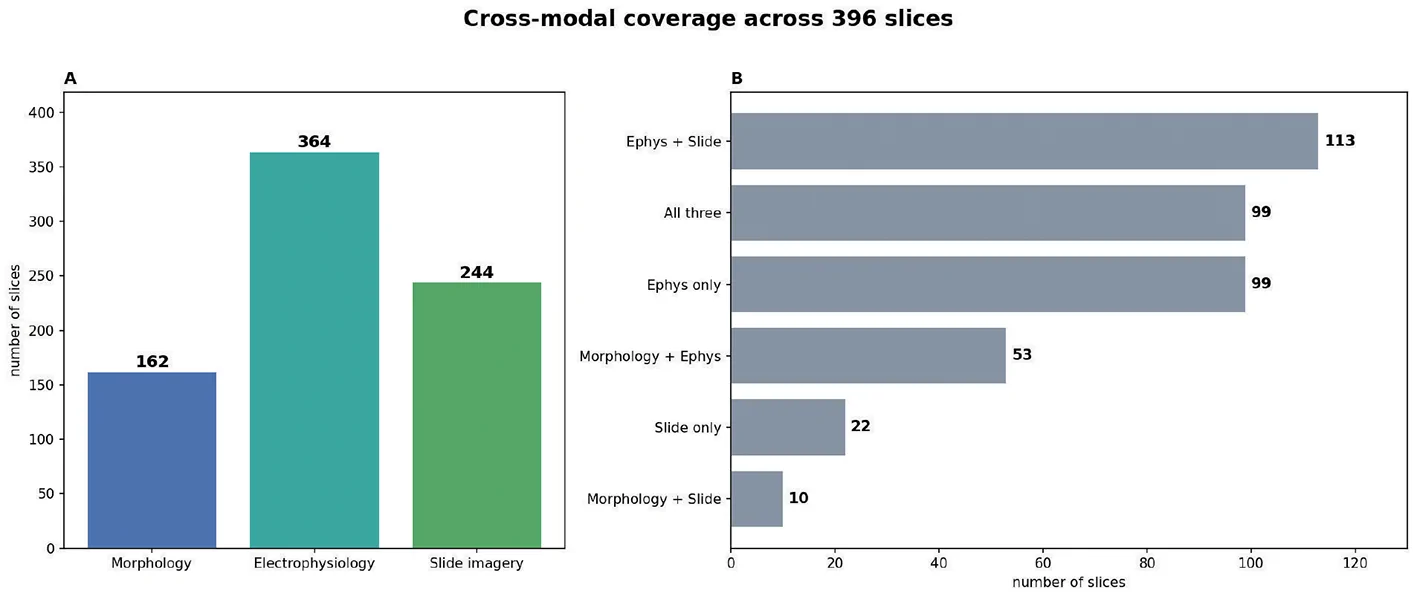
Cross-modal coverage of the dataset: the number of slices carrying each combination of morphology, whole-cell electrophysiology, and histological slide imagery (full per-combination counts are given in the Data Completeness text). **(A)** Slices carrying modality. **(B)** Cross-model combinations per slice.

The composition of the curated cohort and the extent of cross-modal characterization are summarized in Figure 4.

## Discussion

### The value of the dataset

This resource pairs large-scale, standardized intrinsic electrophysiology with detailed single-cell morphology for neocortical neurons, recorded under a single adaptive stimulus protocol applied identically across all cells. The uniform protocol and the preservation of raw signals in community-standard NWB and SWC formats make the data directly suitable for cell-type classification, biophysically detailed single-neuron modeling, and machine-learning studies of the structure–function relationship. Indexing into tidy Parquet tables with a cross-modal join key lowers the barrier to querying and integrating the two modalities at scale.

### The limitations of the dataset

The data reflect the LNMC data gathered by MT and are not a population-representative survey: morphology and electrophysiology are linked for 62 individual cells (spanning the 152 slices that carry both modalities), with the remaining cells characterized in a single modality. ALL morphological reconstructions and electrophysiological recordings are from rat. Certain acquisition-level details (e.g., per-sweep stimulus amplitudes) and some histological and reconstruction parameters were not recorded in the source archives. Brain-region labels resolve to two levels of the source atlas hierarchy: cells localized to the recording site carry ‘Primary somatosensory area, hindlimb representation', while cells for which only the containing structure was recorded carry its parent region ‘Cerebral Cortex'. Anatomical annotation is therefore coarser for the latter group, and no hemisphere or stereotaxic cell-position coordinates were recorded for any cell.

### Opportunities for future research

Future extensions could expand cross-modal coverage by reconstructing additional recorded cells, add extracted electrophysiological feature tables (e.g., firing rate, adaptation, input resistance) computed directly from the NWB sweeps, and incorporate morphometric summaries derived from the SWC trees. The standardized stimulus set also lends itself to automated e-type classification and to fitting and validating biophysical models against the recorded responses.

## Conclusion

This dataset provides a comprehensive, FAIR^2^-aligned, AI-ready resource pairing cortical neuron morphology and intrinsic electrophysiology from the EPFL Laboratory of Neural Microcircuitry. With 244 reconstructions, 641 standardized recordings, canonical NWB and SWC assets, and tidy cross-linked tables, it supports reproducible analysis and machine-learning workflows on the structure and function of neocortical neurons.

## Author's note

This document and the accompanying data package were prepared by the authors with assistance from the Frontiers FAIR^2^ Data Management Platform.

## Data Availability

The dataset, interactive Data Explorer, FAIR^2^ Data Package, methods documentation, and accompanying variable-level data dictionary will become publicly accessible upon publication through the FAIR^2^ Data Portal at https://doi.org/10.71728/senscience.bt07-sboc.
